# OA11 Beyond the classical phenotype: a case series of atypical giant cell arteritis presentations

**DOI:** 10.1093/rap/rkag113.011

**Published:** 2026-11-03

**Authors:** Jonathan Woodward, Ritsuko Saito, Ching Hua Chai, Nikita Goel

**Affiliations:** Queen Alexandra Hospital, Portsmouth, United Kingdom; Queen Alexandra Hospital, Portsmouth, United Kingdom; Queen Alexandra Hospital, Portsmouth, United Kingdom; Queen Alexandra Hospital, Portsmouth, United Kingdom

## Abstract

**Introduction:**

In this four patient case series we will disucss some interesting atypical presentations of GCA. These patients were referred through diverse clinical pathways, each demonstrating distinct diagnostic and therapeutic challenges. They highlight the importance of maintaining clinical suspicion for GCA despite atypical manifestations and emphasise the complementary roles of imaging, histology and multidisciplinary assessment in establishing the diagnosis and guiding management.

**Case description:**

**Case 1**; A 79-year-old man presented with headache, jaw claudication, polymyalgic symptoms and visual disturbance. Despite highly suggestive symptoms, inflammatory markers were normal (CRP 1.9 mg/L, ESR 2 mm/hr) and temporal artery ultrasound was equivocal. Following progression to bilateral anterior ischaemic optic neuropathy (AION), he received intravenous methylprednisolone. Temporal artery biopsy confirmed GCA.

**Case 2**; A 71-year-old woman initially felt to have bilateral vertebral artery dissection, later developing bilateral vertebral artery occlusion and posterior circulation stroke. She lacked classical cranial symptoms and inflammatory markers remained minimally elevated. The progression of vertebral changes led to a concern of GCA. PET-CT demonstrated vertebral artery vasculitis consistent with large-vessel GCA. During glucocorticoid tapering she developed AION despite persistently low inflammatory markers, requiring intravenous methylprednisolone and escalation to tocilizumab.

**Case 3;** A 92-year-old man presented with transient right eyelid drooping and markedly elevated inflammatory markers (CRP 186 mg/L, ESR 67 mm/hr). He denied headache, jaw claudication, scalp tenderness, constitutional symptoms and polymyalgic features. Temporal artery ultrasound demonstrated diffuse arterial wall thickening and PET-CT showed bilateral vertebral artery uptake. He responded successfully to high dose oral glucocorticoid therapy and remains in remission following steroid withdrawal.

**Case 4;** A 65-year-old man presented with weight loss, recurrent vertigo, headaches and migrainous visual phenomena (pixelation and fish scale changes in vision). He denied jaw claudication, scalp tenderness, visual loss and polymyalgic symptoms. Inflammatory markers were elevated (CRP 44 mg/L, ESR >100 mm/hr). Ultrasound demonstrated halo signs in temporal and axillary arteries, confirming cranial and large-vessel vasculitis. MR angiography subsequently identified a cerebellar infarct, while CT angiography demonstrated multifocal intracranial and extracranial arterial stenoses. Treatment was escalated to intravenous methylprednisolone followed by high-dose oral corticosteroids, resulting in biochemical remission.

**Discussion:**

This series demonstrates the broad clinical spectrum of GCA and the limitations of relying on traditional diagnostic features. Presentations ranged from biopsy-proven cranial disease with normal inflammatory markers to large-vessel vasculitis presenting with cerebrovascular complications, unexplained inflammatory syndromes and unusual neurological symptoms.

Two patients developed significant ocular complications despite normal or minimally elevated inflammatory markers, reinforcing that CRP and ESR cannot reliably exclude active disease. In one case, biopsy-proven GCA with bilateral AION occurred despite completely normal inflammatory markers and non-diagnostic ultrasound findings. These observations highlight the need to prioritise clinical judgement when suspicion is high.

Large-vessel involvement was a prominent feature in three cases, affecting the vertebral arteries and resulting in vertebrobasilar pathology including dissection, occlusion and stroke. One patient presented predominantly with recurrent vertigo and positive migrainous visual symptoms rather than classical cranial manifestations, while another presented with transient ptosis and isolated inflammation. These presentations illustrate how GCA may initially mimic neurological, cerebrovascular or general medical conditions.

Advanced imaging proved particularly valuable. PET-CT established the diagnosis in patients lacking classical features and identified clinically occult vertebral artery vasculitis. Vascular ultrasound demonstrated both cranial and extracranial involvement and facilitated rapid treatment initiation. Neurovascular imaging also identified extensive cerebrovascular disease not apparent clinically and directly influenced escalation of immunosuppressive therapy.

Close collaboration between Rheumatology, Neurology, Stroke Medicine, Ophthalmology, Radiology and Haematology was essential is these cases.

Overall, this series reinforces that GCA remains fundamentally a clinical diagnosis. Atypical symptoms, normal inflammatory markers or inconclusive investigations should not exclude consideration of the disease when clinical suspicion remains high.

**Key learning points:**

• An open minded approach is key to not missing atypical cases of GCA. It is a clinical heterogenous disease and the majority of these patients presented without scalp pain, jaw claudication, or visual loss, instead presenting with atypical neurological, cerebrovascular or inflammatory features.

• A major learning point is that normal or minimally elevated inflammatory markers do not exclude active, biopsy-proven or sight-threatening GCA. Two patients developed significant ophthalmic complications despite normal or near-normal CRP and ESR values, emphasising the importance of clinical judgement when suspicion remains high.

• The cases also illustrate the diverse manifestations of large-vessel GCA. Vertebral artery involvement seems particularly difficult to detect early and persistent vertigo is not often taught as a symptom of GCA . In one patient, positive visual phenomena, including migrainous aura were a prominent feature, this is very abnormal for GCA which would normally present with negative eye symptoms such as visual loss.

• These cases also demonstrate the value of complementary imaging modalities. Temporal artery ultrasound enabled rapid diagnosis in some patients, while PET-CT and neurovascular imaging identified clinically occult large-vessel vasculitis and a greater burden of vascular disease than was apparent from clinical assessment alone. Imaging findings directly influenced treatment decisions, including escalation of immunosuppressive therapy. Combining modalities such as MRA and Ultrasound was key in case 4.

• Successful diagnosis and management relied on close collaboration between Rheumatology, Neurology, Stroke Medicine, Ophthalmology, Radiology and Haematology services. In a number of these cases it is our colleagues in other specialties who correctly identified the risk of GCA.

• Overall, these cases reinforce the need to maintain a high index of suspicion for GCA in adults presenting with unexplained neurological symptoms, cerebrovascular events or inflammatory markers, even when classical symptoms are absent or investigations are initially inconclusive.

